# Genetic Crosstalk Between Type 1 Diabetes and Sjögren's Syndrome: A Systematic Exploration of Risk Genes and Common Pathways

**DOI:** 10.1111/jcmm.70930

**Published:** 2025-11-26

**Authors:** Aamir Fahira, Kai Zhuang, Xuemin Jian, Syed Mansoor Jan, Yong Liu, Jianbo Sun, Yongyong Shi, Zunnan Huang

**Affiliations:** ^1^ Dongguan Key Laboratory of Computer‐Aided Drug Design, The First Dongguan Affiliated Hospital Guangdong Medical University Dongguan Guangdong China; ^2^ Guangdong Medical University Key Laboratory of Big Data Mining and Precision Drug Design, Guangdong Provincial Key Laboratory for Research and Development of Natural Drugs, School of Pharmacy Guangdong Medical University Dongguan Guangdong China; ^3^ Bio‐X Institutes, Key Laboratory for the Genetics of Developmental and Neuropsychiatric Disorders (Ministry of Education) Shanghai Jiao Tong University Shanghai China; ^4^ NHC Key Laboratory of Nuclear Technology Medical Transformation, Mianyang Central Hospital, School of Medicine University of Electronic Science and Technology of China Mianyang Sichuan China; ^5^ Department of Bioinformatics and Biostatistics, School of Life Sciences and Biotechnology Shanghai Jiao Tong University Shanghai China

**Keywords:** causal risk genes, genetic pleiotropy, Mendelian randomization, pathway analysis, Sjögren's syndrome, type 1 diabetes

## Abstract

Sjögren's Syndrome (SS) and Type 1 Diabetes (T1D) are autoimmune disorders that can co‐occur in patients, leading to complex clinical presentations. Despite observational evidence of their co‐occurrence, the underlying genetic mechanisms remain poorly understood. To investigate the shared genetic factors and pathways between SS and T1D, we conducted a comprehensive analysis using multiomic approaches. Conditional and conjunctional false discovery rate analyses were performed to identify genetic polygenicity and overlap between the two diseases. Functional annotation and pathway analysis identified SNPs with regulatory potential. Furthermore, Mendelian Randomization (MR) analyses were employed to investigate causal associations between gene expression and disease risk. Single‐cell differential gene expression analysis was also employed to validate the associations of risk genes with T1D and SS. Our analysis identified 36 shared loci, revealing common genetic enrichment between SS and T1D. Functional annotation and pathway analysis revealed 52 credible genes involved in cysteine‐related processes, apoptotic signalling and immune responses. MR analyses revealed that AC007283.5 was positively linked with both SS and T1D, while PLEKHM1 and CRHR1‐T1 were negatively associated. Additionally, CERS2 was positively associated with SS, DEF6 was positively associated with T1D, and KANSL1‐AS1 was negatively associated with T1D, indicating the presence of complex regulatory mechanisms. Moreover, Single‐cell differential gene expression analysis confirmed the dysregulation of risk genes in SS and T1D. This study identified shared genetic factors and pathways underlying SS and T1D, highlighting cysteine‐related processes and apoptotic signalling. The findings underscore the complex interplay of autoimmunity and the need for targeted treatments addressing their common mechanisms.

## 1 Introduction

Type 1 diabetes mellitus (T1D) and Sjögren's syndrome (SS) are both autoimmune disorders that can co‐occur in individuals, often as part of a broader spectrum of autoimmune polyglandular syndromes (APS). T1D can be complicated by the coexistence of other autoimmune disorders, such as SS, and this combination may be indicative of APS, which includes various types of autoimmune conditions [1]. A significant proportion of T1D patients exhibit sicca symptoms, which are characteristic of SS, and these symptoms may be more pronounced during hyperglycemic phases. The presence of antinuclear antibodies and anti‐Ro antibodies in these patients supports the potential co‐occurrence of SS [2, 3]. Hypertriglyceridemia was observed to be significantly higher in patients with primary Sjögren's syndrome compared to the control group (22% vs. 15%, *p* = 0.023) [4]. Furthermore, SS can lead to renal involvement, although it is relatively rare. The most common renal manifestation of SS is tubulointerstitial nephritis, which shares pathological features with the glandular infiltration seen in the syndrome [5]. In addition, electrolyte disturbances, such as hypokalaemia, can be associated with SS and may be particularly relevant in patients with coexisting autoimmune conditions, including T1D [6]. The presence of anti‐SS‐A/Ro and anti‐SS‐B/La antibodies is common in SS, and these may coexist with anti‐glutamic acid decarboxylase (GAD) antibodies, which are also found in autoimmune diabetes mellitus cases [7]. The co‐occurrence of T1D and SS is supported by the presence of shared auto‐antibodies and overlapping clinical features, such as sicca symptoms and autoimmune thyroid disease. The relationship between these conditions is complex and may involve various autoimmune mechanisms, highlighting the importance of comprehensive screening for associated autoimmune disorders in patients presenting with either T1D or SS.

The genetic underpinnings of T1D and SS are complex and interconnected. Both conditions are autoimmune, with T1D affecting insulin production and SS impacting moisture‐producing glands. The non‐obese diabetic (NOD) mouse model has been instrumental in studying the genetic factors, apoptosis, autoantibodies and cytokines involved in the disease's pathogenesis [8, 9]. In NOD mice, IFN‐γ plays a critical role in the early preimmune phase and the later immune phase of SS‐like autoimmune exocrinopathy. Knockout studies of IFN‐γ and its receptor in NOD mice suggest that IFN‐γ is involved in the onset of the disease, affecting acinar cell apoptosis and salivary protein expression [10]. Researchers using the NOD mouse model have provided valuable insights into the genetic and immunological factors contributing to the pathogenesis of SS and its association with T1D [11]. The role of IFN‐γ in the development of SS‐like autoimmune exocrinopathy highlights the complexity of the immune response in these conditions. Additionally, the prevalence of sicca symptoms and specific autoantibodies in T1D patients supports the notion of a shared autoimmune basis between these two diseases [10]. Furthermore, a study based on familial aggregation and heritability revealed that T1D is associated with an increased risk of developing SS [12].

Observational studies have reported the co‐occurrence of T1D and SS; however, the underlying genetics of the shared aetiology of these disorders are still elusive. This study intended to explore the shared underlying genetic factors associated with T1D and SS. Genome‐wide association studies (GWAS) have identified numerous genetic loci associated with T1D and SS, which provide the basis for exploring the common genetic factors of these disorders [13, 14]. A comprehensive genome‐wide association analysis was conducted to investigate the shared genetic factors between T1D and SS. This study used the genetic pleiotropic association analysis via PleioFDR (https://github.com/precimed/pleiofdr) [15] method to explore the common underlying genetics associated with T1D and SS, followed by functional association analysis. Furthermore, to investigate the causal influence of the expression of the predicted gene on both T1D and SS and the bidirectional causal association, we utilised eQTL data of predicted genes sourced from the eQTLGen consortium and GWAS summary statistical data for T1D and SS, employing Mendelian randomization (MR) analysis and colocalization analysis. Moreover, single‐cell differential gene expression analysis was conducted to explore the expression pattern of the risk genes associated with T1D and SS.

## 2 Methods and Materials

### 2.1 Genome‐Wide Association Summary Statistical Datasets

#### 2.1.1 SS GWAS Summary Statistical Data

The GWAS summary statistical dataset of SS was retrieved from a recent large‐sample‐size GWAS study conducted by Khatri et al. [13]. The datasets are publicly accessible at the Database of Genotypes and Phenotypes (dbGaP) under the accession number phs002723.v1.p1. Concisely, Khatri et al. [13] conducted a GWAS with a sample size of 27,610, including 3885 cases and 23,725 controls from the European population to identify the genetic risk factor associated with SS.

#### 2.1.2 T1D GWAS Summary Statistical Data

T1D GWAS summary statistical dataset was obtained from the GWAS catalogue (GCST90014023). Briefly, Joshua Chiou et al. [14] conducted a GWAS study with a large sample size of 520,580, including 18,942 cases and 501,638 controls of European ancestry, and identified 81 loci, including 33 novel loci associated with T1D.

### 2.2 Data Harmonisation

Before analysis, data harmonization analysis was carried out. Briefly, the GWAS summary statistical dataset of SS consists of SNPs rsID, chromosome, position, effect allele, other allele, sample size, case–control information and odds ratio. Firstly, we used the odds ratio to calculate the *β* value, that is,

$$\beta =\log\;\left(\mathrm{odd}\;\text{ratio}\right).$$

For each SNP and then used the *β* value to calculate the standard error, that is,

$$\mathrm{SE}=\text{sqrt}\;\left(\frac{{\left(\beta \right)}^{2}}{\mathrm{qchisq}\;\left(p.\text{value},1,\text{lower}.\text{tail}=F\right)}\right).$$

Since the used software, that is, PleioFDR [15], LDSC (Linkage Disequilibrium Score Regression) [16] and SUPERGNOVA (SUPER Genetic covariance Analyser) [17] required *β*, SE, *Z*‐score, *p*‐value and SNP information as input. Furthermore, the MAF was missing in the summary statistical dataset. We used the approximation of the allele frequency from the 1000 Genomes Phase 3 file as mentioned in the GenomicSEM pipeline (https://github.com/GenomicSEM/GenomicSEM/wiki/2.1‐Calculating‐Sum‐of‐Effective‐Sample‐Size‐and‐Preparing‐GWAS‐Summary‐Statistics) for the SS dataset [18]. Besides, the T1D summary statistical dataset was in hg38 format; to convert the hg38 to hg19 we utilised MungeSumstats (https://github.com/neurogenomics/MungeSumstats) liftover function [19].

### 2.3 Genome‐Wide Polygenic Overlap Analysis of SS and T1D

To investigate the genetic architecture of SS and T1D, MiXeR software (https://github.com/precimed/mixer) was utilised to conduct univariant [20] and bivariant analysis [21]. The MHC region was initially included in the analysis due to its relevance to both autoimmune disorders. However, due to the strong influence of the MHC region, we also conducted the analysis excluding it to assess the results without its impact. Furthermore, independent runs were conducted to ensure robust standard deviation values. Analysis parameters were determined and visualised using log‐likelihood plots and tables generated by MiXeR [20, 21].

### 2.4 Genetic Correlation and Local Genetic Covariance Analysis

Before performing the common genetic association analysis, we conducted the global genetic correlation analysis via LDSC (https://github.com/bulik/ldsc) [16]. It allows us to explore the shared genetic basis between SS and T1D by leveraging summary statistics from GWAS. LDSC takes advantage of the patterns of linkage disequilibrium (LD) in the genome to estimate genetic correlations across a wide range of traits. Furthermore, to predict the local genetic covariance, we used the SUPERGNOVA (https://github.com/qlu‐lab/SUPERGNOVA), a statistical method to predict specific genomic region overlap across the SS and T1D [17].

### 2.5 Identification of Shared Genetic Variants

Conditional and conjunctional false discovery rate (FDR) analyses were used to find the common genetic loci (https://github.com/precimed/pleiofdr) [15]. Concisely, conditional FDR (condFDR) improves the efficiency of SNP statistical association analysis by recalculating the statistical significance of genetic variations for the main trait via leveraging cross‐trait SNP enrichment. CondFDR analysis was performed twice on each trait as the primary phenotype to enhance SNP prediction power. Subsequently, conjunctional FDR (conjFDR) analysis was used to identify common genetic variants based on the two condFDR results. ConjFDR value was defined as the largest value between the condFDRs for both phenotypes, providing estimates for identifying shared genetic variants. This analysis excluded the MHC region to understand the shared genetic enrichment across the traits because the MHC region was previously implicated in SS and T1D. Furthermore, the *p*‐threshold for condFDR was retained at 0.01, and for conjFDR at 0.05, as suggested by Andreassen et al. [15].

### 2.6 Functional Annotation Analysis of Predicted Shared SNPs

To elucidate the shared underlying genetic aetiology, a functional annotation analysis was conducted on the identified shared SNPs. Concisely, using the default parameters, Functional Mapping and Annotation (FUMA) was utilised to identify the independent genetic loci and the leading single‐nucleotide polymorphism (SNP conjFDR < 0.05) [22]. Furthermore, the Combined Annotation Dependent Depletion (CADD) [23], RegulomeDB [24] and chromatin states [25] approaches were used to conduct the functional association analysis for all predicted shared SNPs within at least one independent SNP in LD *r* ^2^ (0.6) [26]. FUMA gene mapping methods were employed to identify candidate genes associated with SS and T1D. These methods included physical gene mapping, eQTL analysis and Hi‐C chromatin interaction approaches to predict the candidate genes [22]. Furthermore, to investigate the underlying mechanisms of the predicted genes, we then utilised the g:GOST module of the gprofile server (https://biit.cs.ut.ee/gprofiler/gost) [27]. This server performs functional enrichment analysis, also known as over‐representation analysis (ORA) or gene set enrichment analysis, on the provided gene list. It maps genes to established functional information sources, including the Human Phenotype Ontology [28], known annotations from the pathway databases of KEGG [29], Reactome [30] and WikiPathways [31], CORUM (the comprehensive resource of mammalian protein complexes) [32] and protein–protein interactions, and predicts regulatory relations from TRANSFAC [33] and miRTarBase [34] to identify statistically significant enriched terms.

### 2.7 Mendelian Randomization Analysis

#### 2.7.1 Bidirectional MR Analysis

To explore the bidirectional causal relationship between SS and T1D, we conducted MR analyses in both directions. SNPs associated with SS and T1D were selected at genome‐wide significance with a *p*‐value < 5 × 10^−8^. For SS, 11 SNPs were retained after clumping and removing palindromic and ambiguous SNPs. For T1D, 55 SNPs were selected, following similar quality control procedures.

Multiple MR methods were used to estimate the causal effects, including Inverse‐Variance Weighted (IVW), MR Egger, Weighted Median, Simple Mode and Weighted Mode [35]. These methods allow for robust estimation under different assumptions regarding pleiotropy and instrument strength. Heterogeneity was assessed using Cochran's *Q* statistic, which provides insight into the variability among the SNPs used as instruments. The presence of pleiotropy was evaluated using the MR Egger intercept. Furthermore, the MR‐pleiotropy residual sum and outlier (MR‐PRESSO) method was used to remove influential outliers, producing raw and outlier‐corrected estimates [36]. This comprehensive approach ensures that the causal inferences made are robust to potential biases and pleiotropic effects. Furthermore, we used a *p*‐value < 1 × 10^−5^ for IV (instrumental variable) selection and reran the whole analysis.

### 2.8 The Causal Influence of the Predicted Gene Expression on SS and T1D

#### 2.8.1 Retrieving Expression Quantitative Trait Loci (eQTL) Data

The eQTL data have been widely employed to detect genetic variations that influence gene expression and are essential in establishing connections between exposures and outcomes in MR studies. Therefore, the eQTL dataset obtained from blood tissues was included in the MR study. The dataset utilised in this study was obtained from the eQTLGen consortium (https://eqtlgen.org/). This dataset consists of 31,684 blood tissue samples, predominantly derived from European populations [37]. The samples included in the dataset were selected based on their statistical significance (*p*‐value < 5 × 10^−8^), with an FDR below 0.05.

#### 2.8.2 Data Preparation for MR Analysis

Before conducting the MR analysis, essential data preprocessing steps were performed. Briefly, the eQTLs dataset and GWAS data were harmonised to account for additive genetic effects. For eQTL datasets, SNPs within ±5 kb of predicted gene regions were selected for cis effects and precision. To mitigate LD bias, significant SNPs (*p*‐value < 5 × 10^−8^, FDR < 0.05) were clumped with an LD threshold of *r* ^2^ = 0.001, using European cohorts from the 1000 Genome project as the reference panel via Plink. These preprocessing measures collectively establish a robust foundation for rigorous MR analysis by enhancing data quality and minimising confounding factors.

#### 2.8.3 MR Analysis of Risk Genes, and SS and T1D

MR analysis, which uses genetic variations as instrumental variables (IVs), assists in determining the validity of a putative causal effect link between an exposure and an outcome. This is dependent on the random distribution of genetic variations during reproduction. A two‐sample MR analysis was employed using the R package TwoSampleMR (version 0.5.4) (https://mrcieu.github.io/TwoSampleMR/) [35]. The Wald ratio was utilised to evaluate the causal influence of predicted genes if they had only one accessible SNP after data processing. Furthermore, IVW was used for genes with two or more accessible SNPs. IVW integrates Wald ratio values from individual SNPs into one causal estimate for each risk factor. A weighted median analysis was performed on genes with more than two available SNPs to calculate the median of weighted SNP Wald ratio estimates. To prevent the possibility of a type 1 error, the Bonferroni correction method was employed with a Bonferroni‐corrected *p*‐value threshold of 0.05/number of genes. To assess the robustness of the results, sensitivity analyses were conducted, including the weighted median method and MR‐Egger regression [35], and MR‐PRESSO analysis [36]. Sensitivity analyses allow for the exploration of potential violations of the MR assumptions, such as horizontal pleiotropy or violations of the exclusion restriction assumption.

### 2.9 Colocalization Analysis of Significant MR Results

For a two‐sample MR analysis, the same SNP must link the gene association and outcome, that is, SS and T1D. However, instances can arise where distinct SNPs independently influence the gene and SS and T1D. To address this, we performed colocalization analysis by employing the R package coloc (https://www.rdocumentation.org/packages/coloc/versions/5.1.0) [38] to identify shared causal variants. This analysis yielded five hypotheses (H0–H4), with H4 indicating a single shared SNP influencing both traits. We mainly considered H4, and its associated posterior probability (PP) approximates the likelihood of a shared causal variant impacting both factors. We retained SNPs that were strongly supported (PP. H4 > 0.7) and their corresponding genes. Genes showing significance in MR analysis and passing colocalization were considered potential causal risk genes for SS and T1D [38].

### 2.10 Single‐Cell Differential Gene Expression Analysis of Risk Genes

The scRNA‐seq datasets for SS and T1D were obtained from the Gene Expression Omnibus (GEO) database [39] with accession numbers GSE157278 [40] and GSE239501 [41]. The R package ‘Seurat’ [42], as a comprehensive toolkit for single‐cell RNA sequencing (scRNA‐seq), was utilised to process the scRNA‐seq data. Initially, Seurat objects were created for each dataset, with a filtering criterion of a minimum of three detected cells and 50 expressed features to ensure data quality, and cells with more than 25% mitochondrial content or fewer than 50 features were excluded to eliminate potential low‐quality or stressed cells. The data were then normalised using the ‘LogNormalize’ method. The ‘FindNeighbors’ and ‘FindClusters’ functions were then used for clustering the cells. Clusters were visualised using t‐distributed stochastic neighbour embedding (t‐SNE) [43]. Cell type annotation was carried out using the SingleR package [44], which accurately assigns cell types to each cluster.

### 2.11 Drug‐Target Gene Enrichment Analysis

The Drug Signatures Database (DSigDB; http://dsigdb.tanlab.org/DSigDBv1.0/) links drugs and compounds to target genes, facilitating gene set enrichment analysis [45]. DSigDB encompasses 22,527 gene sets, representing 17,389 unique compounds associated with 19,531 genes. The Enrichr platform (https://maayanlab.cloud/Enrichr/) provides a web‐based tool to analyse these connections, utilising DSigDB to identify drugs with potential relevance based on gene enrichment [46]. Compounds with an adjusted *p*‐value below 0.05 are considered statistically significant.

### 2.12 Summary of Workflow

**FIGURE 1:**
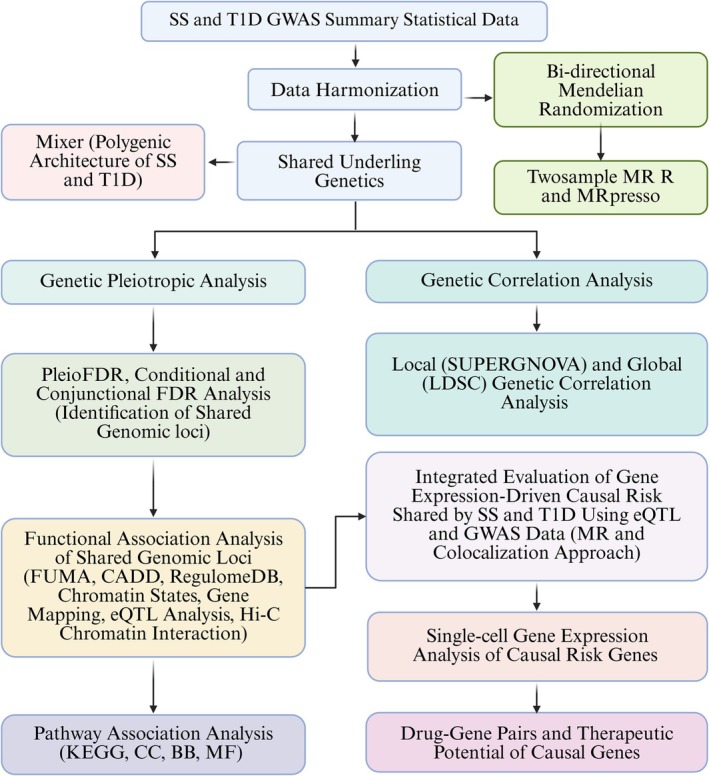
Step‐by‐step flow‐work conducted in this study.

As shown in Figure 1, the study employed a comprehensive approach to explore the shared genetic architecture and potential causal relationships between SS and T1D using GWAS summary statistics. Concisely, the summary statistical data were retrieved for SS and T1D, and data harmonisation was conducted to ensure compatibility and consistency across datasets for unified analysis. Next, the shared underlying genetics between SS and T1D was investigated through a comprehensive genome‐wide association analysis. Briefly, polygenic architecture was assessed using the MiXeR tool, identifying loci contributing to both diseases. Genetic correlation analysis was conducted using LDSC and SUPERGNOVA, revealing genetic correlations. Additionally, genetic pleiotropy analysis was used to determine specific genetic loci that influenced both diseases. This study also investigated potential causal relationships between SS and T1D using MR analysis. This bidirectional causal analysis employed two‐sample MR and MR‐PRESSO methods to assess the directionality and strength of the causal relationships while correcting for potential pleiotropy and outliers. The shared genomic loci identified were subjected to functional association analysis to predict causal genes and the underlying pathways involved in SS and T1D. This analysis was followed by MR analysis using eQTL data to explore the causal risk genes. Single‐cell gene expression analysis was performed to explore the expression pattern of risk genes associated with SS and T1D. Moreover, to identify potential drugs that interact with the predicted genes, we analysed drug–gene associations and evaluated their relevance to the targeted conditions. This integrated methodological approach allowed for a robust exploration of the genetic connections between SS and T1D, providing insights into their shared genetic foundations and potential causal links.

## 3 Results

### 3.1 Genetic Polygenicity of SS and T1D

#### 3.1.1 Impact of Shared MHC Genetic Susceptibility Between T1D and SS

**FIGURE 2:**
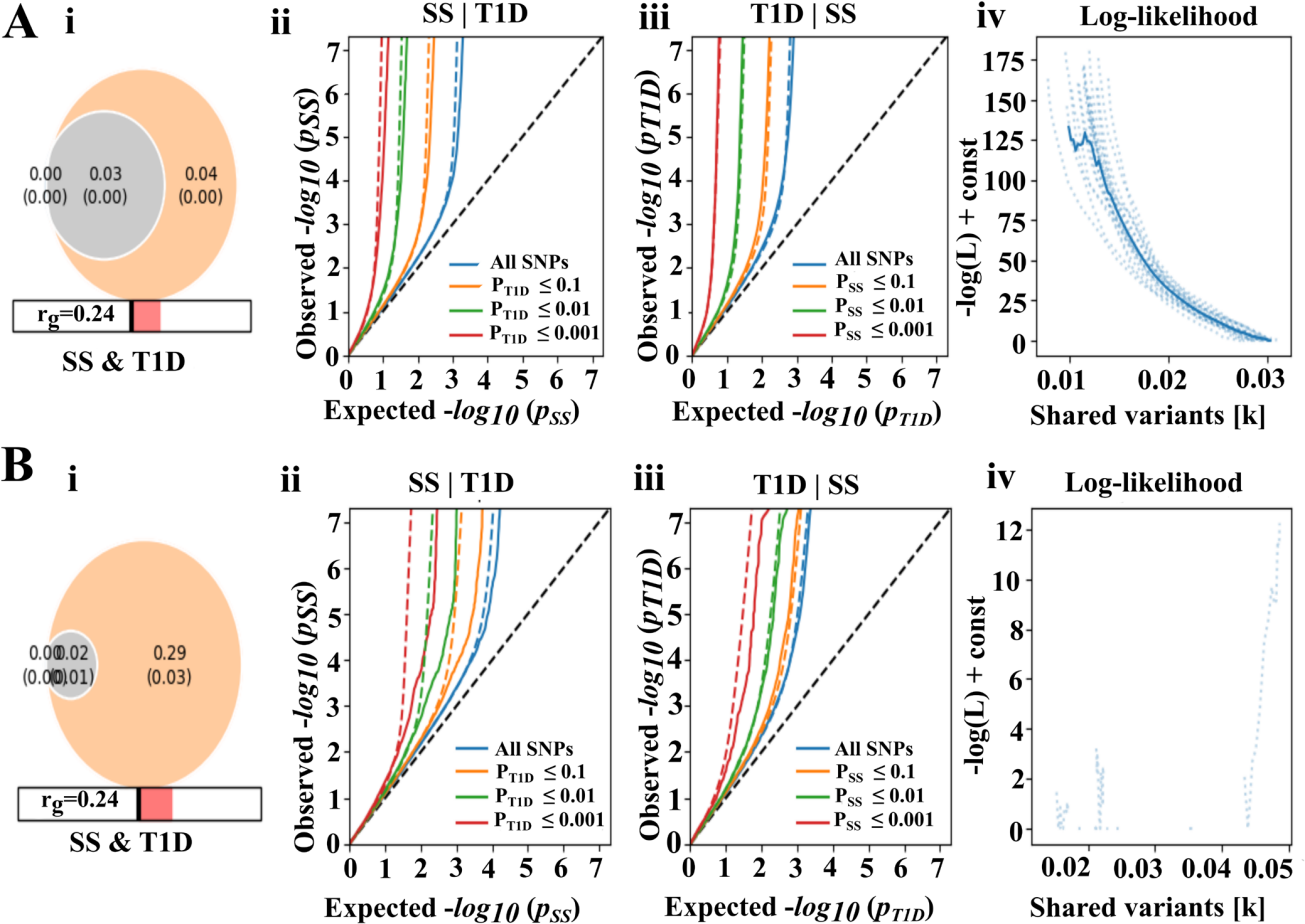
Genetic polygenicity and shared variants between T1D and SS analysed using MiXeR software. **(A)** Results including the MHC region. (B) Results excluding the MHC region. (i—A, B) Venn diagrams illustrating trait‐specific and shared polygenic components, with genetic correlation (*r*_*g*_) below. (ii–iii—A, B) Q‐Q plots showing observed versus expected −log10(*p*) values for both traits conditional on the other. SNPs are stratified by significance thresholds. (iv—A, B) Log‐likelihood curves as a function of shared variant number *k*.

Figure 2 depicts the genetic relationship between T1D and SS using MiXeR, comparing results when the MHC region is included (Panel A) versus excluded (Panel B). The Venn diagrams in A(i) show that a modest proportion of variants are shared between T1D and SS, with a slightly lower genetic correlation (*r*_*g*_ = 0.24) when the MHC region is included (Figure 2A (i)) compared to when it is excluded (*r*_*g*_ = 0.26, Figure 2B (i)). The Q‐Q plots ii and iii (A, B) demonstrate significant deviations from the expected distribution under the null hypothesis of no association. This indicates that the shared genetic architecture between the two diseases is enriched for highly significant loci. In the log‐likelihood plots iv (A, B), the inclusion of the MHC region indicates a stronger fit for shared variants when this region is considered. Excluding the MHC region shifts the model, showing that although polygenicity remains, the MHC region plays a substantial role in shared genetic liability between T1D and SS.

#### 3.1.2 Genetic Polygenicity and Global Genetic Overlap of SS and T1D Excluding MHC

Despite minimal overall genetic correlation, the univariate and bivariate MiXeR analyses reveal a partial polygenicity between SS and T1D. Concisely, the univariate MiXeR analysis excluding the MHC region predicted positive AIC (Akaike Information Criterion) and BIC (Bayesian Information Criterion) values, which indicate that the summary statistical data have the power to apply the MiXeR model; however, the bivariate MiXeR analysis showed the minimum possible polygenicity between SS and T1D (Table S1 MiXeR). Furthermore, the LDSC analysis revealed that SS and T1D are genetically correlated with a *p*‐value of 0.0002 (Table S2 LDSC). Figure 2B represents the genetic polygenicity predicted between SS and T1D, excluding the MHC region. Briefly, Figure 2B(i) shows the genetic correlation, Figure 2B (ii and iii) depict the genetic overlap between SS and T1D, and Figure 2B (iv) illustrates the partial likelihood. The MiXeR analysis predicts substantial shared SNPs and genetic correlation between SS and T1D. In conclusion, the MiXeR bivariate analysis identified 23 shared genetic variants between SS and T1D (Table S1 MiXeR). Local genetic correlation analysis revealed that the chromosome 1 region chr1:112561693–114451307 and chromosome 11 region chr11: 333347–1057948 are highly correlated within SS and T1D with local genetic correlation values of 0.99 (*p*‐value = 5.395 × 10^−6^) and 1.12 (*p*‐value = 3.67 × 10^−5^), respectively (Table S3 supergenova).

#### 3.1.3 Conditional and conjunctional FDR Results

**FIGURE 3:**
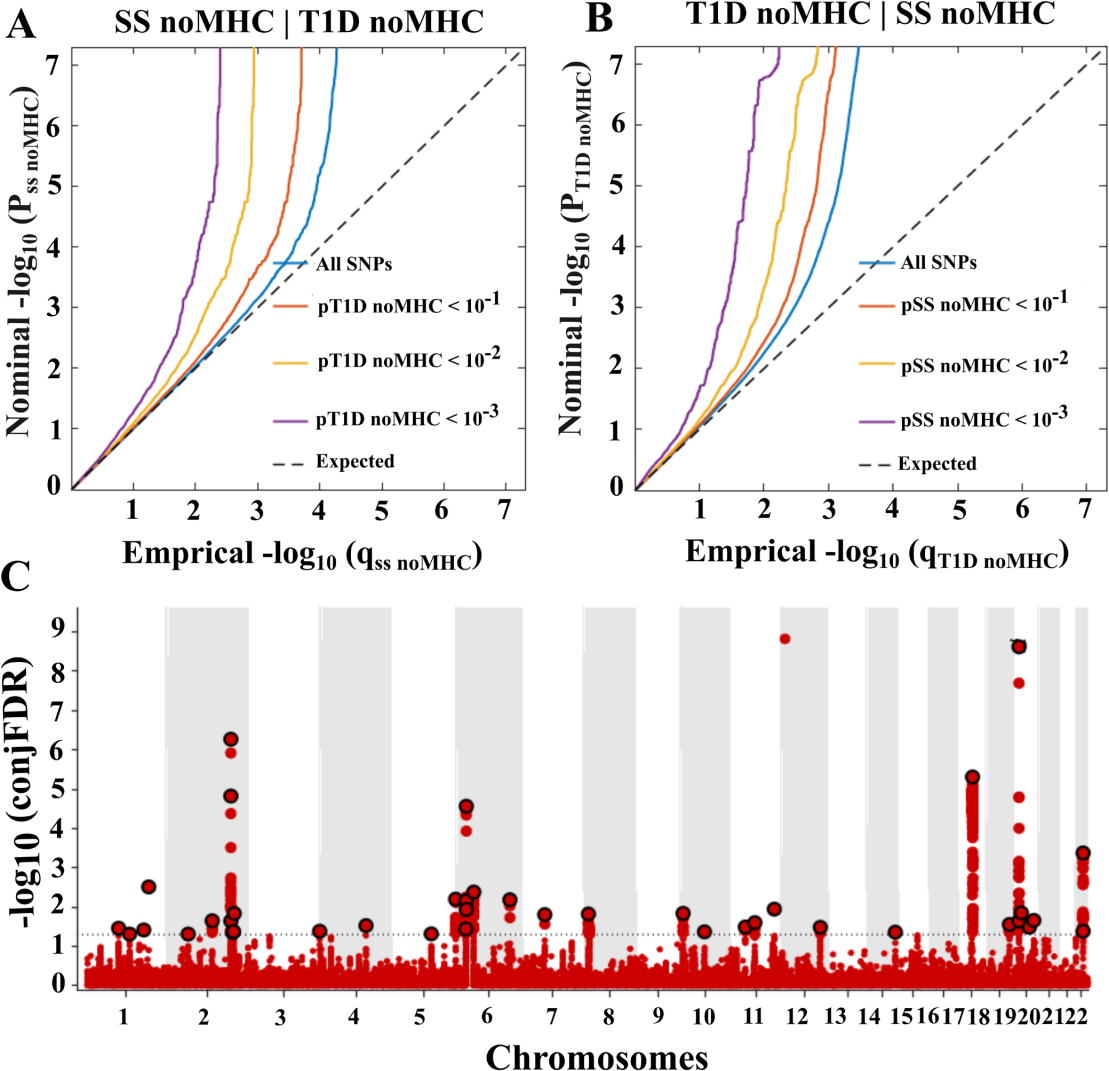
Conditional and conjunctional FDR analysis. (A, B) depict the Q‐Q plot of the expected and observed relation between the SS and T1D, respectively. Different colours represent the nested strata for all shared SNPs between the two traits, that is, SNPs *P*cond_phenotype < 0.1 (red), *P*cond_phenotype < 0.01 (orange) and *P*cond_phenotype < 0.001 (purple). The dashed black line illustrates the anticipated pattern when there is no association. As the significance of the SNPs in the conditional phenotype increases, a greater deviation toward the left from the no‐association line suggests polygenic overlap. (C) represents the conjFDR‐predicted loci associated with both SS and T1D.

Figure 3 illustrates the conditional Q‐Q plots for SS and T1D, displaying a pronounced enrichment in both directions. Briefly, a significant leftward shift was observed among the group of SNPs with higher significance (Figure 3A,B), suggesting genetic enrichment and potential shared genetic background between SS and T1D. Upon applying condFDR to analyse both traits, we found that it exhibited comparable power to unconditional FDR analysis, indicating its effectiveness in controlling false discoveries while maintaining statistical power. Overall, 36 shared loci were predicted by conjFDR analysis (Figure 3C).

### 3.2 Functional Annotation and Pathway Association Analysis

**FIGURE 4:**
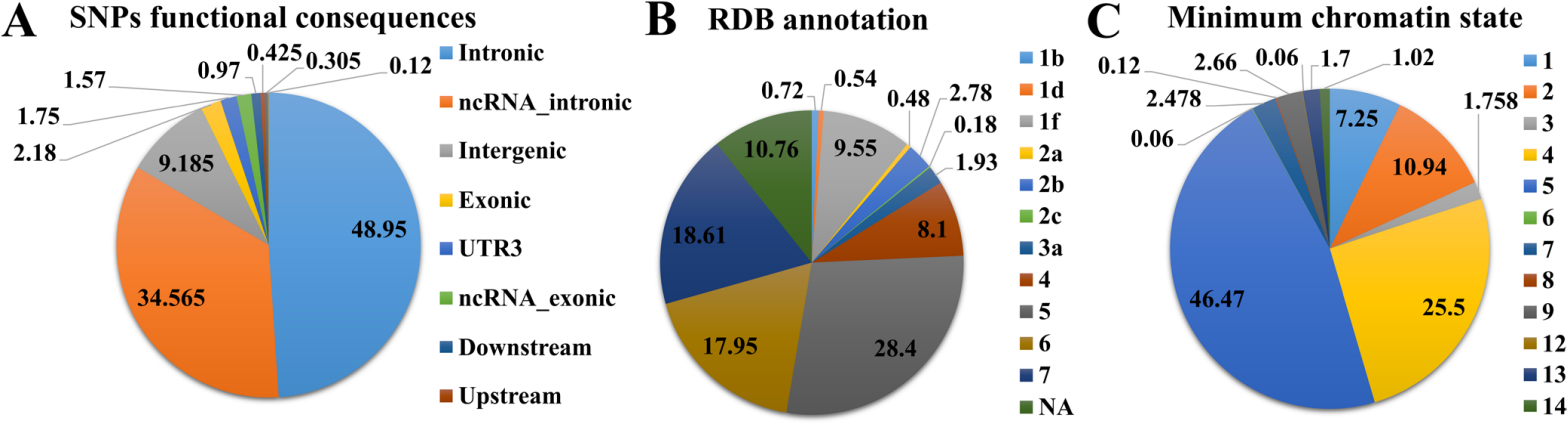
Functional annotation of predicted shared loci associated with T1D and SS. (A) Depicts the functional consequences of the SNPs identified. (B) Presents the prioritisation of SNPs based on RegulomeDB scores, indicating their regulatory potential. (C) displays the minimum chromatin state of the predicted loci.

**FIGURE 5:**
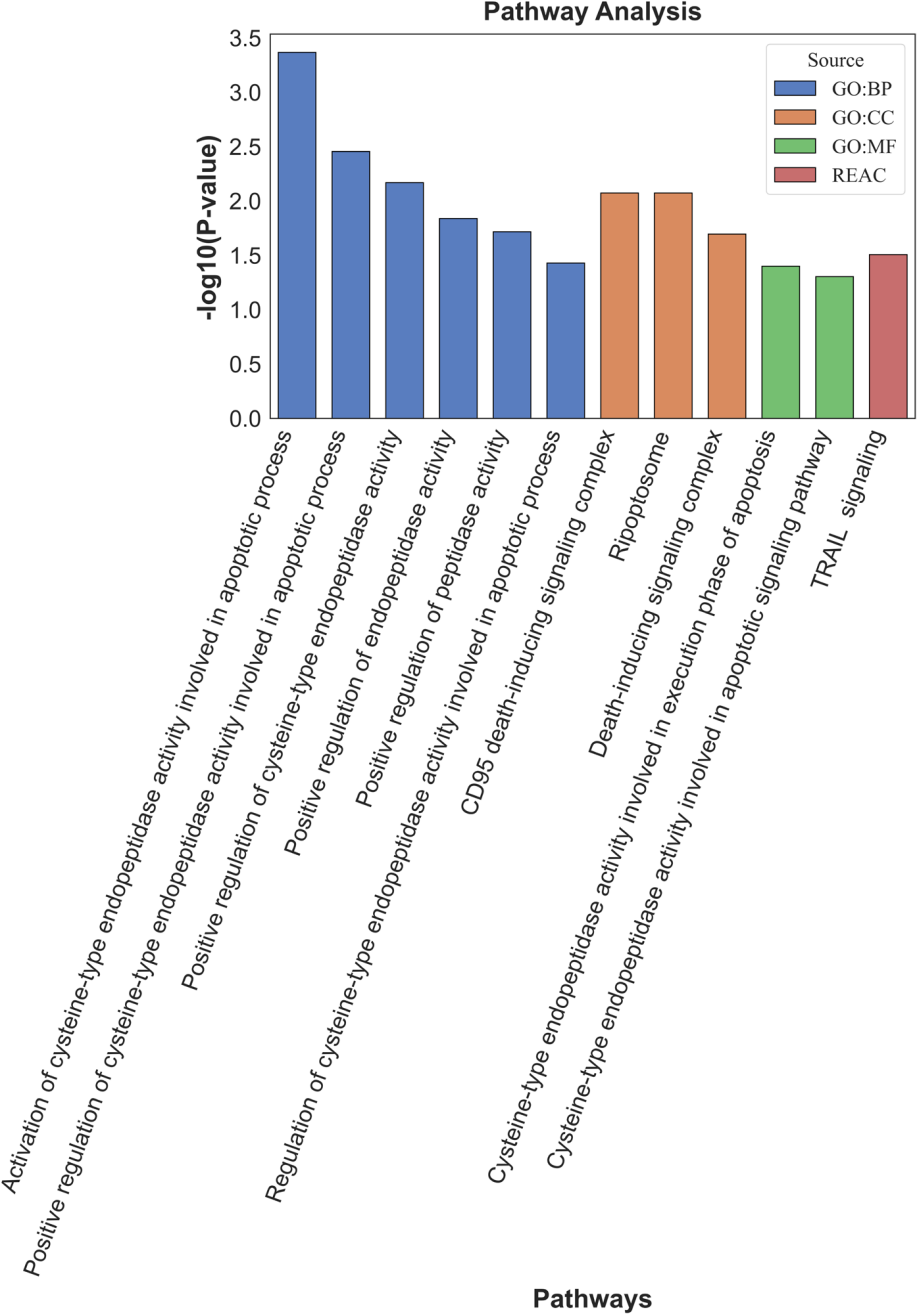
Pathway association analysis. Pathways associated with the combined pathophysiology of SS and T1D, highlighting the complex interactions and molecular mechanisms involved in both conditions.

One thousand six hundred fifty six SNPs were found in the LD with 36 shared loci associated with SS and T1D, functional prediction and gene association analysis via FUMA predicted highly prioritised SNPs and credible genes (Table S4 SNPs). Concisely, the functional consequences analysis revealed that 48.95% of SNPs were in the intronic region (Figure 4A), while the regulomeDB annotation results showed that 16.18% were highly functional SNPs (Figure 4B). Similarly, 95.46% of SNPs were in the open chromatin region (minimum chromatin states ≤ 7) (Figure 4C). The CADD score revealed that 59 SNPs were highly deleterious (Table S4 SNPs). The functional annotation analysis identified highly functional SNPs associated with SS and T1D (Table S5 Functional annotation). FUMA gene mapping analysis predicted 52 highly prioritised and credible genes (Table S6 FUMA_genes). These credible genes were used to predict the shared pathways associated with the shared aetiology of SS and T1D. Concisely, pathway association analysis of predicted credible genes identified important gene sets involved in Biological Process (BP), Molecular Function (MF) and Cellular component (CC), that is, cysteine‐type endopeptidase activity involved in the apoptotic signalling pathway, execution phase of apoptosis, CD95 death‐inducing signalling complex, and ripoptosome (Figure 5). More details are given in Table S7.

### 3.3 Mendelian Randomization Analysis

#### 3.3.1 Bidirectional Causal Relation Between SS and T1D

##### 3.3.1.1 SS on T1D

The MR analysis exploring the causal effect of SS on T1D provided significant evidence of a causal relationship (Table 1). The IVW method yielded a *β* coefficient of 0.480 (SE = 0.125, *p*‐value = 1.24 × 10^−4^), suggesting a strong positive association. The MR Egger method indicated an even stronger effect with a *β* of 0.784 (SE = 0.172, *p*‐value = 1.4 × 10^−3^). The Weighted Median method also supported this association (*b* = 0.284, SE = 0.048, *p‐*value = 3.88 × 10^−9^). However, the Simple Mode and Weighted Mode methods did not produce significant results. Significant heterogeneity was detected in both the MR Egger and IVW analyses (*Q*‐statistics = 499.8, *p*‐value = 6.29 × 10^−102^ for MR Egger; *Q* = 772.4, *p‐*value = 1.77 × 10^−159^ for IVW), indicating variability among the SNPs. The MR Egger intercept suggested potential pleiotropy (intercept = −0.142, SE = 0.064, *p‐*value = 0.054). MR‐PRESSO identified 10 influential outliers, emphasising the need for cautious interpretation, though these outliers did not substantially alter the overall findings. Specific SNPs, such as rs11085727 and rs11889341, exhibited strong associations with SS and T1D, highlighting genetic overlaps (Tables S8–S9). The results remained consistent when applying a *p*‐threshold of < 1 × 10^−^^5^ (Table 1).

**TABLE 1:** Bidirectional causal relationship of SS and T1D.

MR analysis		SNPs	Method	Causal estimate (*β*, SE, *p*)	Heterogeneity (*Q*, df, *p*)	Pleiotropy (Intercept, SE, *p*)	MR‐PRESSO outlier‐corrected causal estimate (*β*, SE, *p*)
SS on T1D	Exposure with *p* < 5 × 10^−8^	11	IVW	0.48, 0.125, 1.24 × 10^−4^	772.4, 10, 1.77 × 10^−159^	‐0.142, 0.064, 0.054	0.458, 0.06, 0.088
			MR‐Egger	0.784, 0.172, 1.4 × 10^−3^	499.8, 9, 6.29 × 10^−102^		
			Weighted median	0.284, 0.048, 3.38 × 10^−9^			
T1D on SS		55	IVW	0.015, 0.052, 0.773	294.6, 54, 7.60 × 10^−35^	0.0199, 0.015, 0.214	0.104, 0.031, 1.71 × 10^−3^
			MR‐Egger	−0.054, 0.075, 0.479	286.1, 53, 1.08 × 10^−33^		
			Weighted median	0.077, 0.049, 0.115			
SS on T1D	Exposure with *p* < 1 × 10^−5^	42	IVW	0.330, 0.065, 4.19 × 10^‐7^	1345.6, 41, 1.62 × 10^−255^	−0.109, 0.025, 1.26 × 10^−4^	0.160, 0.033, 5.47 × 10^−5^
			MR‐Egger	0.674, 0.097, 2.62 × 10^−8^	927.9, 40, 1.48 × 10^−168^		
			Weighted median	0.047, 0.027, 0.079			
T1D on SS		122	IVW	0.058, 0.039, 0.133	439.3, 121, 1.09 × 10^−37^	0.026, 0.008, 2.82 × 10^−3^	0.118, 0.029, 1.17 × 10^−4^
			MR‐Egger	‐0.062, 0.056, 0.232	407.8, 120, 5.18 × 10^−33^		
			Weighted median	0.081, 0.048, 0.088			

##### 3.3.1.2 T1D on SS

The analysis of T1D's causal effect on SS showed less consistent evidence (Table 1). The IVW method reported a non‐significant association with a *β* coefficient of 0.015 (SE = 0.052, *p*‐value = 0.773), and the MR Egger method indicated a negative but non‐significant effect (*β* = −0.054, SE = 0.075, *p*‐value = 0.479). The Weighted Median method hinted at a potential association (*β* = 0.077, SE = 0.049, *p*‐value = 0.115), but only the Weighted Mode method suggested a significant effect (*β* = 0.090, SE = 0.038, *p*‐value = 0.023). Significant heterogeneity was observed in the MR Egger (*Q*‐statistic = 286.1, *p*‐value = 1.08 × 10^−33^) and IVW (*Q* = 294.6, *p*‐value = 7.60 × 10^−35^) analyses. The MR Egger intercept did not show significant pleiotropy (intercept = 0.019, SE = 0.015, *p*‐value = 0.214). Importantly, MR‐PRESSO correction of outliers led to a significant causal estimate (*β* = 0.104, SE = 0.031, *p*‐value = 1.71 × 10^−3^), suggesting that outliers may have masked a potential causal relationship. SNPs such as rs1002985 and rs112162078 were significantly associated with both T1D and SS, indicating possible shared genetic risk factors (Tables S10–S11). The results were consistent when a *p*‐threshold of < 1 × 10^−5^ was applied (Table 1).

### 3.4 Causal Risk of Expression of Predicted Genes With SS and T1D

**FIGURE 6:**
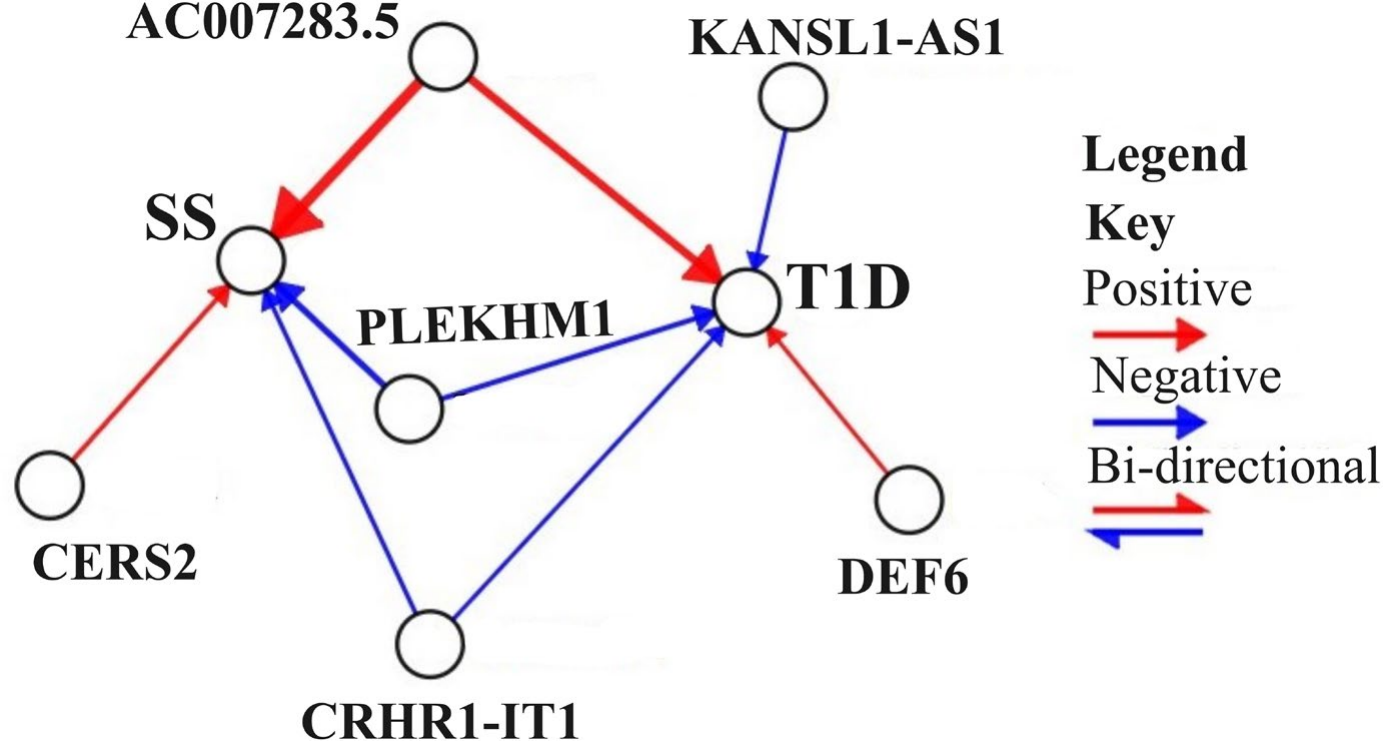
The causal association of predicted genes with SS and T1D. Red indicates a positive causal relationship between the genes (exposure) and the outcomes (SS, T1D), while blue indicates a negative relationship.

MR analysis was conducted to explore the causal relationship between the expression of the predicted genes and SS and T1D. Cis‐eQTL data were retrieved for predicted genes from eQTLGen phase 1 (https://www.eqtlgen.org/cis‐eqtls.html). Concisely, data for 30 genes, encompassing 448 SNPs, were available and used as exposures. LD clumping was employed to remove SNPs within the LD region utilising the European reference panel, resulting in 65 SNPs associated with 30 genes, which were then used as the exposure for the MR analysis. This methodological approach allowed us to investigate the potential causal effects of the expression of predicted genes on SS and T1D, leveraging the genetic variants as instrumental variables. To prevent the possibility of a Type 1 error, the Bonferroni correction method was employed. Specifically, a Bonferroni‐corrected *p*‐value threshold of 0.05/30, resulting in a cutoff value of 1.61 × 10^−3^, was selected as a statistically significant relationship. For T1D, five genes were found to have causal risk associations. Among them, AC007283.5 (ENSG00000234431, *β* = 0.0595, SE = 0.0153 and *p*‐value = 9.91 × 10^−5^), and DEF6 (ENSG00000023892, *β* = 0.0024, SE = 0.0007 and *p*‐value = 6.73 × 10^−4^) were positively linked with T1D. On the other hand, KANSL1‐AS1 (ENSG00000214401, *β* = −0.0033, SE = 0.0006, *p*‐value = 7.74 × 10^−8^), CRHP1‐IT1 (ENSG00000204650, *β* = −0.0026, SE = 0.0005 and *p*‐value = 7.05 × 10^−8^) and PLEKHM1 (ENSG00000225190, *β* = −0.0165, SE = 0.0026, *p*‐value = 7.83 × 10^−10^) were negatively associated with T1D (Figure 6). Furthermore, four genes were found to have causal risk for SS; interestingly our analysis revealed that AC007283.5 (ENSG00000234431, *β* = 0.0962, SE = 0.0296 and *p*‐value = 1.17 × 10^−3^) and CERS2 (ENSG00000143418, *β* = 0.0096, SE = 0.0028 and *p*‐value = 6.30 × 10^−4^) were positively associated with SS, while CRHR1‐IT1 (ENSG00000204650, *β* = −0.0063, SE = 0.0011 and *p*‐value = 2.74 × 10^−9^) and PLEKHM1 (ENSG00000225190, *β* = −0.0329, SE = 0.0059 and *p*‐value = 3.32 × 10^−8^) were negatively associated with SS (Figure 6). Table 2 and supplementary Tables S12–S13 represents the MR analysis results. MR results were visualised with MR‐Automatic Network Arranger (MiRANA) (https://github.com/CMorenoStokoe/network‐mr‐vis‐tool).

**TABLE 2:** Mendelian Randomization Results for Predicted Genes associated with SS and T1D.

Outcome	Exposure	Method	nsnp	*β*	SE	*p*
SS	PLEKHM1	Wald ratio	1	−0.0329	0.0059	3.32 × 10^−8^
SS	AC007283.5	Wald ratio	1	0.0962	0.0296	1.17 × 10^−3^
SS	CERS2	Wald ratio	1	0.0096	0.0028	6.30 × 10^−4^
SS	CRHR1‐IT1	Wald ratio	1	−0.0063	0.0011	2.74 × 10^−9^
T1D	PLEKHM1	Wald ratio	1	−0.0165	0.0027	7.83 × 10^−10^
T1D	AC007283.5	Wald ratio	1	0.0595	0.0153	9.91 × 10^−5^
T1D	DEF6	Wald ratio	1	0.0024	0.0007	6.73 × 10^−4^
T1D	KANSL1‐AS1	Wald ratio	1	−0.0033	0.0006	7.74 × 10^−8^
T1D	CRHR1‐IT1	Wald ratio	1	−0.0026	0.0005	7.05 × 10^−8^

### 3.5 Colocalization Analysis

Table 3 presents the colocalization analysis results that support the MR findings. H4 > 0.8 demonstrates strong colocalization, while H4 < 0.8 shows moderate colocalization. For DEF6, the colocalization analysis results (PP.H4 = 0.356) indicate a moderate association. Concisely, the colocalization analysis highlights the genetic overlap between T1D, SS and risk genes. The table displays exposure, that is, risk genes used as exposure for colocalization analysis, the number of SNPs analysed (nsnps), as well as the posterior probabilities (PP) for five hypotheses: H0.ab (no association with either trait), H1.ab (association with the exposure only), H2.abf (association with the outcome only), H3.abf (independent association with both traits) and H4.abf (shared association with both traits and outcome SS and T1D).

**TABLE 3:** Colocalization analysis of MR significant results.

Exposure/Gene	nsnps	PP.H0.ab	PP.H1.ab	PP.H2.abf	PP.H3.abf	PP.H4.abf	Outcome
PLEKHM1	1	0	0	3.19 × 10^−5^	0	0.999	SS
AC007283.5	1	0	0	0.277	0	0.722	SS
CERS2	1	0	0	0.306	0	0.694	SS
CRHR1‐IT1	1	0	0	3.04 × 10^−6^	0	0.999	SS
PLEKHM1	1	0	0	3.45 × 10^−6^	0	0.999	T1D
AC007283.5	1	0	0	0.126	0	0.873	T1D
KANSL1‐AS1	1	0	0	3.26 × 10^−4^	0	0.999	T1D
DEF6	1	0	0	0.643	0	0.356	T1D
CRHR1‐IT1	1	0	0	3.15 × 10^−4^	0	0.999	T1D

### 3.6 Single‐Cell Differential Gene Expression Analysis of Risk Genes

**FIGURE 7:**
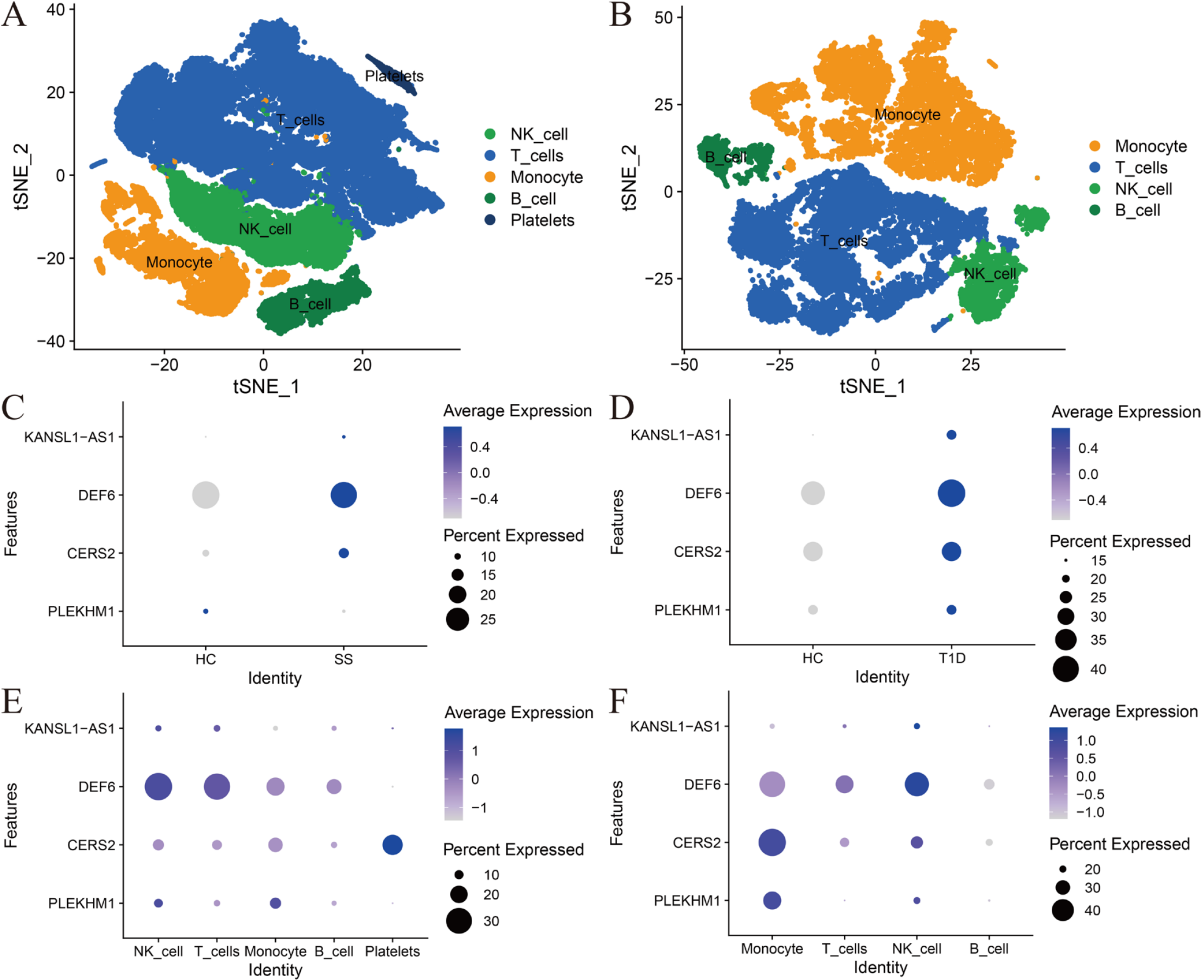
Single‐cell differential gene analysis of risk genes in SS and T1D. (A, B) depict the identified 5 and 4 types of cells in SS and T1D, respectively. Among them, green represents NK cells, blue represents T cells, orange represents monocytes, dark green represents B cells and dark blue represents platelets. (C, D) represent the expression of. KANSL1‐AS1, DEF6 and CERS2 in SS and T1D disease and control samples. (E, F) represent the expression of KANSL1‐AS1, DEF6 and CERS2 in various cells of SS and T1D.

The single‐cell differential gene expression analysis identified five cell types in SS, including NK cells, T cells, monocytes, B cells and platelets (Figure 7A). In T1D, four cell types were also identified, comprising NK cells, T cells, monocytes, and B cells (Figure 7B). In SS, KANSL1‐AS1, DEF6 and CERS2 are upregulated, while PLEKHM1 is downregulated (Figure 7C). In T1D, KANSL1‐AS1, DEF6, CERS2 and PLEKHM1 are all upregulated (Figure 7D). Moreover, in both SS and T1D, KANSL1‐AS1 is upregulated in NK cells and T cells, DEF6 is upregulated in NK cells, T cells and monocytes, CERS2 is upregulated in NK cells, monocytes and PLEKHM1 is upregulated in NK cells and monocytes (Figure 7E,F). These findings suggest that the expression of these four genes is consistent between SS and T1D.

### 3.7 Drug‐Gene Pairs and Therapeutic Potential for Targeted Conditions

**FIGURE 8:**
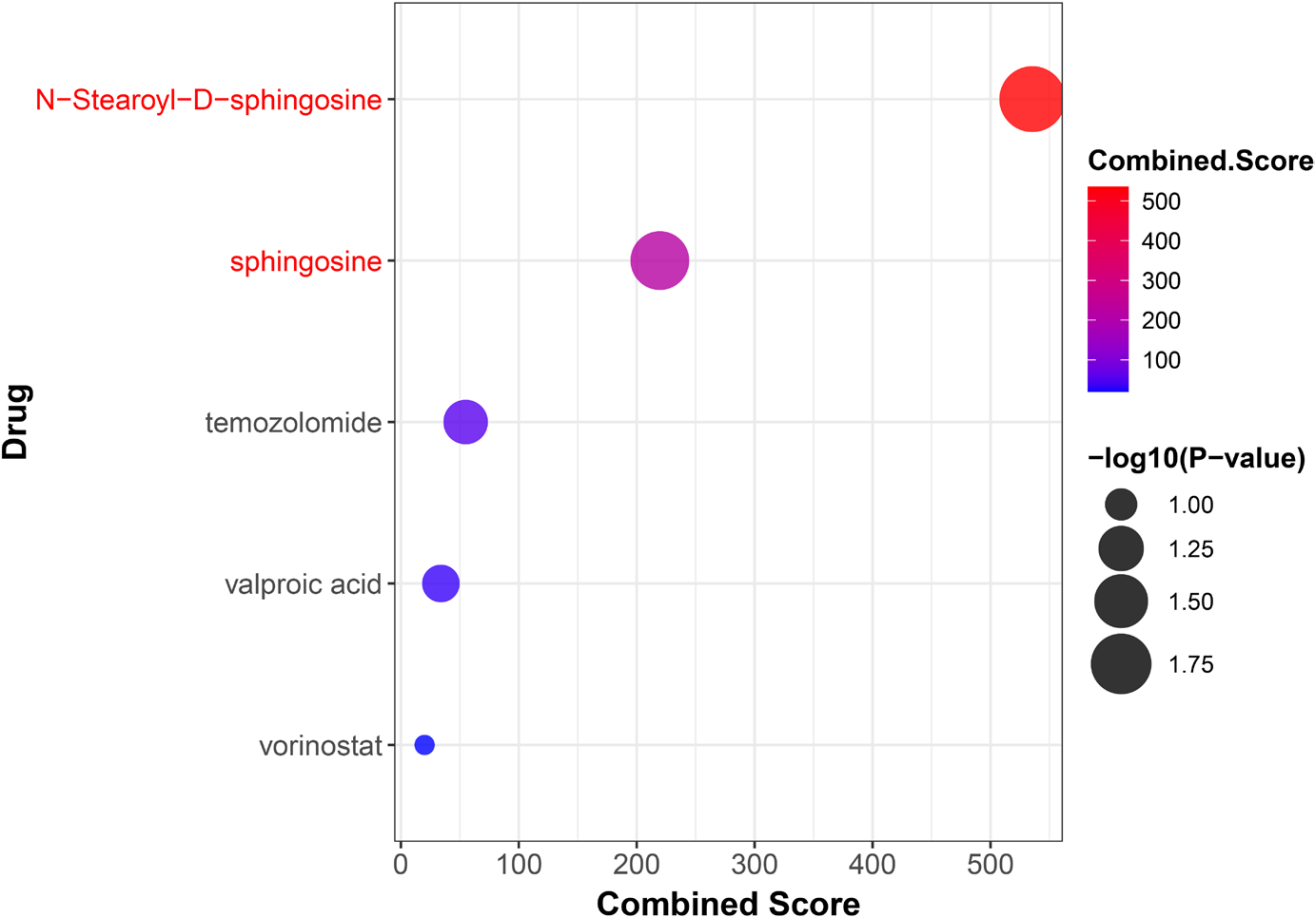
Candidate drugs ranked by combined score and significance, with N‐Stearoyl‐D‐sphingosine and sphingosine identified as top candidates for targeting CERS2.

The analysis identified 43 significant drug‐gene pairs, as detailed in Table S14. Among these, N‐Stearoyl‐D‐sphingosine and sphingosine were promising compounds targeting the CERS2 gene, achieving statistical significance (*p*‐value < 0.05), as illustrated in Table 4 and Figure 8. These compounds are particularly noteworthy due to their therapeutic relevance, as they show potential for treatment applications in both SS and T1D.

**TABLE 4:** The top five predicted drugs and their corresponding genes.

Drug	*p*	Odds ratio	Combined score	Genes
N‐Stearoyl‐D‐sphingosine	0.010	117.412	535.473	CERS2
Sphingosine	0.021	56.926	219.608	CERS2
Temozolomide	0.060	19.499	54.966	DEF6
Valproic acid	0.084	13.685	33.955	PLEKHM1
Vorinostat	0.119	9.436	20.125	PLEKHM1

## 4 Discussion

This study provides an in‐depth exploration of the shared genetic mechanisms underlying the co‐development of SS and T1D, two autoimmune disorders with complex etiologies. Utilising large‐scale GWAS data, the conjFDR method was applied to uncover common genetic loci associated with the risk of both SS and T1D. Subsequent pathway enrichment and MR analyses highlighted critical regulatory genes, such as those involved in cysteine‐type endopeptidase activity, which may play a central role in mediating the risk of both SS and T1D. These findings advance our understanding of the genetic crosstalk between these two autoimmune diseases and may guide future therapeutic strategies aimed at targeting shared biological pathways.

This study revealed the shared underlying pathways that contribute to the co‐development of SS and T1D, offering insight into common mechanisms influencing both diseases. Concisely, the pathway ‘cysteine‐type endopeptidase activity’ emerged as significantly associated with both conditions. This pathway is integral to T1D pathophysiology through multiple facets: the presence of core cysteine residues in islet autoantigens can affect autoantibody binding, reduced cysteine‐dependent antioxidative defenses contribute to β‐cell oxidative stress, and cysteinyl leukotriene signalling can modulate insulin secretion, thereby linking inflammatory mediators to metabolic function [47, 48, 49]. Cysteine proteases, such as lysosomal cathepsins, are known to facilitate antigen processing and presentation, a crucial step in immune recognition, and their dysregulation can lead to aberrant immune attacks on self‐tissue [50]. Excessive cysteine protease activity can also promote tissue damage by degrading extracellular matrix and triggering apoptotic pathways [50]. Indeed, studies in a murine model of SS have shown that elevated cysteine protease activity in the salivary glands coincides with the onset of autoimmune glandular inflammation and damage [51]. These observations underscore how protease‐mediated processes, for example, antigen processing and cell death, are a common feature of autoimmune pathophysiology in both T1D and SS, potentially driving the exposure of self‐antigens and the destruction of target tissues.

Another prominent shared mechanism identified is dysregulated apoptotic signalling via death receptor pathways. The CD95 (Fas) death‐inducing signalling complex (DISC), which mediates extrinsic apoptosis, appears to play a role in both SS and T1D [52]. In T1D, pancreatic β cells express the Fas receptor and can undergo apoptosis upon engagement by Fas ligand on infiltrating T cells, contributing to β‐cell loss [53, 54]. The formation of the CD95 DISC involves recruitment of adaptor proteins like FADD and procaspase‐8 (FLICE), whose activation is tightly regulated by factors such as c‐FLIP to prevent excessive cell death. Disruptions in this pathway can have profound effects on immune homeostasis: Fas‐mediated cell death is one of the mechanisms by which cytotoxic lymphocytes kill their targets and also a means to turn off immune responses once pathogens or autoreactive cells are cleared [55]. Alterations in the Fas/FasL system are well known to lead to autoimmune phenomena [56]. For instance, loss‐of‐function mutations in FAS (as in autoimmune lymphoproliferative syndrome) fail to delete autoreactive lymphocytes and cause systemic autoimmunity [55]. Conversely, overactivation of Fas on target cells can cause unwarranted tissue destruction. Our evidence suggests that the Fas DISC is active in both SS and T1D, initiating apoptotic cascades through FADD, procaspase‐8 and procaspase‐10 (with c‐FLIP as a crucial modulator) [55, 57, 58, 59]. This imbalance in Fas‐mediated apoptosis could contribute to autoimmunity in two ways: insufficient apoptosis of immune cells (permitting autoreactive cell survival) and excessive apoptosis of secretory tissue cells (β cells in the pancreas, epithelial cells in salivary glands). Understanding these mechanisms provides valuable insight into potential therapeutic targets, for example, modulating Fas signalling, to restore the proper balance of cell death and survival in both SS and T1D.

Furthermore, TRAIL (TNF‐related apoptosis‐inducing ligand) signalling appears to have a complex, but pivotal, role in the pathophysiology of these autoimmune diseases [60]. Intriguingly, our analysis and prior studies indicate that TRAIL may play a protective role in T1D [61]. Unlike other pro‐inflammatory TNF family cytokines, TRAIL has shown beneficial immunomodulatory effects in T1D: blocking or genetically deleting TRAIL in NOD mouse models accelerates and exacerbates diabetes, whereas administering exogenous TRAIL or increasing TRAIL expression mitigates disease development [61, 62]. Correspondingly, individuals with T1D have been found to exhibit reduced levels of circulating TRAIL, particularly around disease onset, suggesting a deficiency of this protective apoptotic signal during the autoimmune attack on β cells [61]. Mechanistically, TRAIL is thought to protect pancreatic β cells and modulate immune responses by inducing apoptosis preferentially in activated effector T cells and other pathogenic immune cells, thereby preventing excessive autoimmune‐mediated tissue damage [61, 63]. Consistent with this, experimental administration or overexpression of TRAIL in diabetic models has been shown to preserve β‐cell mass and improve outcomes by dampening autoimmunity [64, 65]. These evidences highlight the potential of TRAIL as a therapeutic target for preventing and treating T1D. Moreover, TRAIL signalling plays a significant role in the pathogenesis of SS by inducing apoptosis in salivary gland epithelial cells through its receptors TRAIL‐R1 and TRAIL‐R2 [66, 67]. Notably, the inflammatory cytokine interferon‐γ (IFN‐γ), often elevated in autoimmune lesions, can sensitise salivary gland cells to TRAIL by upregulating TRAIL‐R1 on their surface [67]. This cytokine‐driven enhancement of TRAIL responsiveness is counterbalanced by the presence of decoy receptors (TRAIL‐R3/4 or DCRs), which can sequester TRAIL and inhibit apoptosis. Indeed, SS patients show an imbalance in which pro‐apoptotic TRAIL‐R1/R2 are elevated in affected glands while decoy receptors are low [68, 69]. Additionally, innate immune activation via Toll‐like receptors (e.g., TLR3 sensing viral RNA) has been linked to the apoptosis of glandular cells and chronic inflammation in SS [70]. Viral infections or endogenous nucleic acids engaging TLR3 can trigger local production of type I interferons, which in turn promote lymphocyte infiltration and apoptotic damage in salivary tissues [70]. Taken together, these insights highlight that extrinsic apoptotic pathways and innate immune signals are a shared aspect of SS and T1D pathophysiology. An imbalance in death receptor signalling—whether it be a deficiency of protective signals like TRAIL in T1D or an excess of pro‐apoptotic signalling in target organs as seen with Fas and TRAIL in SS—can tip the scale toward autoimmune tissue destruction. Therapeutically, this suggests that carefully modulating these pathways (for instance, enhancing TRAIL signalling or tempering Fas/TRAIL‐mediated cytotoxicity in target tissues) could be a strategy to protect against tissue damage in both diseases.

Moreover, the two‐sample MR approach was employed to estimate the impact of the expression of predicted genes on the occurrence of SS and T1D. This estimation may determine the most effective drug targets for both diseases. The MR analysis computed the *p*‐value for each gene, which indicated the likelihood of a causal relationship between the expression of the predicted gene and the development of SS and T1D. Briefly, the MR analysis and colocalization analysis identified high‐risk genes associated with SS and T1D, including DEF6, CERS2, KANSL1‐AS1, PLEKHM1, CRHR1‐IT1 and AC007283.5. The single‐cell differential gene expression analysis further supports these findings by demonstrating that genes, that is, DEF6, CERS2 and KANSL1‐AS1, are upregulated in both SS and T1D, and PLEKHM1 is downregulated in SS and upregulated in T1D. The consistency of gene expression patterns across different cell types, including NK cells, T cells, monocytes, B cells and platelets, highlights the shared cellular mechanisms underlying these conditions. Moreover, CRHR1‐IT1 and AC007283.5 were not detected in single‐cell differential expression analysis, likely due to limitations in detection sensitivity or potential methodological constraints. This emphasises the need for further studies to elucidate their possible roles in these diseases.

The DEF6 gene, positively linked to T1D, shows a moderate colocalization threshold (PP.h4.abf value = 0.355). DEF6 plays a key role in activating small GTPases, promoting calcium signalling and T‐cell adhesion. It is also involved in the formation of immunological synapses, as well as T‐cell differentiation and proliferation. In studies on murine autoimmunity, DEF6 deficiency has shown variable effects depending on the genetic background and the specific model of autoimmune disease [71, 72]. DEF6 inhibits the differentiation of both osteoclasts and osteoblasts through a shared mechanism involving feedback inhibition mediated by endogenous type‐I IFN. It serves as a crucial upstream regulator of IFNβ and ISG expression in osteoblasts [73]. Moreover, the CERS2 gene, which is positively linked to SS, is important for producing very long‐chain ceramides and plays a key role in TNF‐R1 internalisation and downstream signalling [74]. Knockdown of the CERS2 gene has been shown to prevent TNF‐R1 internalisation, downstream signalling and glucose‐intolerant behaviour by inhibiting Akt activation in CERS2 knockout mice [75]. Additionally, the KANSL1‐AS1, which is negatively linked to T1D, shows widespread colocalization of its eQTL with meta‐analysis and UKBB data across various epithelial, immune and endothelial cell types [76]. Furthermore, previous research has suggested that the negatively associated gene PLEKHM1 regulates autophagosome‐lysosome fusion, which is involved in controlling selective and nonselective autophagy pathways [77]. The CRHR1‐IT1, a negatively linked gene in T1D and SS, is a spliced transcript variant of CRHR1. The CRHR1 protein is crucial for regulating neuroendocrine, behavioural and autonomic stress responses [78]. When CRHR1 and the GHRH receptor are activated in pancreatic islets, they stimulate insulin synthesis and alter glucocorticoid balance, which may contribute to the development of diabetes mellitus [79]. Finally, although the long noncoding RNA AC007283.5 was not validated in the single‐cell differential gene expression analysis, it has a positive association with both T1D and SS and is linked to interferon‐upregulated pathways. This suggests its involvement in immune responses common to both conditions. Interferon pathways are vital in autoimmune disorders like T1D and SS, regulating inflammation and immune function. Moreover, More et al. [80] and Min et al. [81] revealed that interferon (IFN)‐induced lncRNA AC007283.5 acts as an interferon (IFN)‐stimulated gene and is involved in the innate immune response. AC007283.5's role underscores the shared genetic basis and immune dysregulation seen in individuals with T1D and SS [81].

These findings significantly advance our understanding of the shared pathophysiology of SS and T1D. By identifying key regulatory genes and pathways such as cysteine‐type endopeptidase activity, CD95 death‐inducing signalling complex and TRAIL signalling, this study highlights the multifaceted role of these processes in the development and progression of both diseases. The insights into how cysteine‐related processes, apoptotic signalling and immune responses contribute to the pathogenesis of SS and T1D provide valuable information for identifying potential therapeutic targets. The use of GWAS, MR and pathway analysis has revealed significant genetic overlaps and mechanistic connections, which underscore the complex interplay between autoimmunity and cellular stress responses in these conditions. By elucidating these common mechanisms, this research paves the way for the development of more targeted and effective treatments for individuals suffering from these chronic autoimmune diseases.

Despite the comprehensive integrative genomic and transcriptomic analyses conducted in this study, several limitations must be acknowledged. First, this investigation is entirely computational, and although bioinformatics analyses provide strong predictive insights, the absence of in vitro or in vivo functional validation limits the direct translational applicability of these findings. Functional studies, such as gene knockout models, cellular assays or CRISPR‐mediated gene editing, will be essential in the future to experimentally confirm the roles of the identified genes (e.g., AC007283.5, DEF6, PLEKHM1, CERS2, CRHR1‐IT1 and KANSL1‐AS1) in the pathogenesis of T1D and SS. Second, our analysis utilised GWAS datasets derived exclusively from individuals of European ancestry. While this ensures population homogeneity and reduces confounding, it also restricts the generalisability of the findings to other populations. Given the documented genetic heterogeneity in autoimmune diseases across ethnic groups, future studies incorporating more diverse multi‐ethnic cohorts are crucial to capture population‐specific risk loci and to broaden the applicability of the identified shared mechanisms. Third, the complex and heterogeneous nature of autoimmune diseases, including gene–gene interactions, gene–environment interplay and epigenetic modifications, may not be fully captured in our analyses. Autoimmune disorders such as T1D and SS often involve dynamic and tissue‐specific immune responses that go beyond genetic susceptibility alone. Integrating additional multi‐omics layers, such as proteomics, epigenomics and metabolomics, along with longitudinal clinical data, will help unravel the full spectrum of pathophysiological mechanisms underlying these diseases. Finally, the pathway analyses presented here identify critical immune‐related processes such as cysteine metabolism, apoptotic signalling (CD95 DISC) and TRAIL signalling, which are well‐recognised in the context of autoimmune pathophysiology. However, additional mechanistic studies focusing on how these pathways converge functionally in specific immune cell subsets (e.g., NK cells, T cells, B cells) in the context of T1D and SS will provide deeper mechanistic insight. Future work may benefit from single‐cell multi‐omics technologies to dissect cell‐type‐specific regulatory networks driving these shared autoimmune phenotypes.

## 5 Conclusion

This study elucidates the shared genetic architecture and fundamental pathways between SS and T1D. Through genome‐wide association analysis and functional annotation, we identified 36 common loci and key regulatory genes implicated in both diseases. Pathway analysis highlighted significant roles for apoptosis‐related processes and immune signalling pathways, such as cysteine‐type endopeptidase activity and the CD95 death‐inducing signalling complex. These shared genetic factors and pathways underscore the interconnected nature of these autoimmune disorders and suggest potential targets for therapeutic intervention. Understanding these common mechanisms can enhance the screening and management of patients with co‐occurring SS and T1D, ultimately improving clinical outcomes.

## Author Contributions

**Aamir Fahira:** conceptualization (equal), formal analysis (equal), methodology (equal), visualization (equal), writing – original draft (equal). **Kai Zhuang:** methodology (equal), validation (equal). **Xuemin Jian:** data curation (equal). **Syed Mansoor Jan:** data curation (equal). **Yong Liu:** investigation (equal), validation (equal). **Jianbo Sun:** investigation (equal), validation (equal). **Yongyong Shi:** project administration (equal), supervision (equal), writing – review and editing (equal). **Zunnan Huang:** conceptualization (equal), project administration (equal), supervision (equal), writing – original draft (equal), writing – review and editing (equal).

## Conflicts of Interest

The authors declare no conflicts of interest.

## Supporting information


**Table S1:** Results from MiXeR model estimating polygenicity, effect size variance, and heritability for Type 1 Diabetes (T1D) and Sjögren's Syndrome (SS) GWAS datasets excluding the MHC region.
**Table S2:** Genetic correlation estimates between T1D and SS obtained using linkage disequilibrium score regression (LDSC) analysis.
**Table S3:** Cross‐trait covariance and genetic overlap analysis between T1D and SS using the SUPERGNOVA model.
**Table S4:** Genome‐wide significant SNPs identified in T1D and SS GWAS datasets after quality control and clumping.
**Table S5:** Functional annotation of risk SNPs to assess potential regulatory impact.
**Table S6:** Gene‐based association results from FUMA analysis integrating positional mapping, eQTL, and chromatin interaction data.
**Table S7:** GO and KEGG pathway enrichment analysis of shared genes between T1D and SS using g:Profiler.
**Table S8:** Curated dataset for the MR analysis of SS VS T1D.
**Table S9:** MR results of SS VS T1D.
**Table S10:** Curated dataset for the MR analysis of T1D VS SS.
**Table S11:** MR results of T1D VS SS.
**Table S12:** Mendelian Randomization results showing the causal genes for Sjögren's Syndrome.
**Table S13:** Mendelian Randomization results showing the causal genes for Type 1 Diabetes.
**Table S14:** Drug–gene interaction analysis for shared risk genes using the Drug–Gene Interaction Database (DGIdb).

## Data Availability

The datasets used in this study are publicly accessible. The GWAS summary statistics for Type 1 Diabetes can be found in the GWAS Catalogue under accession number GCST90014023 (https://www.ebi.ac.uk/gwas/). Similarly, the summary statistics for Sjögren's Syndrome are available in the Database of Genotypes and Phenotypes (dbGaP) with the accession number phs002723.v1.p1 (https://www.ncbi.nlm.nih.gov/projects/gap/cgi‐bin/study.cgi?study_id=phs002723.v1.p1).
